# A study on endovascular treatment alone and bridging treatment for acute ischemic stroke

**DOI:** 10.1186/s40001-022-00966-8

**Published:** 2023-01-07

**Authors:** Xiyang Ji, Bo Song, Hao Zhu, Zhao Jiang, Feng Hua, Sa Wang, Jianbo Zhou, Lin Li, Changfei Dai, Mijuan Zhang, Dong Wei, Lele Zhang, Xiaojie Zhang, Qun Zhang, Ping Chen

**Affiliations:** 1 Department of Neurology, Xianyang Hospital of Yan’an University, No. 38 Wenlin Road, Xianyang, 712000 China; 2 Department of Neurology, First Hospital of Xianyang, No. 10, Biyuan Road, Xianyang, 712000 China; 3 Department of Neurology, Xijing Hospital, Air Force Military Medical University, No.169, Changle West Road, Xi’an, 710032 China

**Keywords:** Artificial intelligence, Endovascular treatment, Mechanical thrombectomy, Bridging treatment, Acute ischemic stroke

## Abstract

**Objectives:**

To investigate whether intravenous thrombolysis (IVT) with alteplase (a recombinant tissue plasminogen activator, rt-PA) before endovascular treatment (EVT) is beneficial for acute ischemic stroke (AIS) patients in different periods.

**Methods:**

This study enrolled a total of 140 patients hospitalized between 2019 and 2022 with AIS from large vessel occlusion (LVO) in the anterior circulation. Those patients were divided into the EVT alone group and IVT + EVT group, in which EVT was preceded by intravenous rt-PA. According to the time from onset to femoral artery puncture, the above two groups were divided into the following subgroups: < 4.5 h, between 4.5 and 6 h, between 6 and 8 h, and between 8 and 10 h. There were 78 patients in the EVT alone group and 62 patients in the IVT + EVT group.

**Results:**

There was no statistically significant difference in functional independence, recanalization rate, favorable outcome rate, or mortality between the EVT and IVT + EVT groups (*P* > 0.05). After adjusting for confounding factors, a lower incidence of intracerebral hemorrhage was observed in the EVT group (*P* < 0.05). A comparison of time-dependent efficacy between the two groups showed that within 6–8 h, there were statistically significant differences between admission and postoperation in the National Institutes of Health Stroke Scale scores at 24 h (*P* = 0.01) or 7 days (*P* = 0.02).

**Conclusions:**

Although there was no difference in clinical efficacy and safety between the abovementioned two groups, treatment with IVT + EVT could increase the risk of bleeding compared to EVT. Moreover, in the 6–8 h subgroup, the efficacy of EVT alone was better than that of IVT + EVT.

## Introduction

Stroke is the leading cause of death and disability among adults in China, with high rates of incidence, disability, mortality, and recurrence. The incidence of cerebrovascular events in China is predicted to be approximately 50% higher in 2030 than in 2010 [1, 2]. AIS is the most common type of stroke, accounting for approximately 80% of all strokes [3]. The key to treating AIS is to unclog the obstructed arteries as early as possible [4]. Timely IVT with rt-PA can recanalize the occluded vessels, thereby salvaging the ischemic semidark zone area and ultimately reducing the rates of death and disability caused by ischemic stroke. However, the limited lysis effect of IVT on larger, proximally located thrombi has been reported; in particular, partial lysis may fragment the target thrombus or cause it to migrate distally [5, 6]. Therefore, IVT may also increase the risk of cerebral hemorrhage. To overcome the disadvantage of IVT, more attention has been paid to EVT within the same time frame, which has become the current mainstream treatment option for AIS.

Based on national and international guidelines [7, 8], IVT should be administered before endovascular intervention for treating AIS patients who are eligible for both IVT and EVT. In addition, several randomized clinical trials have consistently shown that patients with LVO in the anterior circulation may benefit from EVT after IVT treatment [9–11]. However, a comparison of clinical profiles, including procedural, clinical outcomes, and safety, between EVT alone and IVT+EVT has not shown a significant difference between those two therapies, even after adjusting for confounding factors [12]. Recently, a published randomized clinical trial (DIRECT-MT) of EVT with or without IVT in AIS patients revealed that EVT alone was not inferior to EVT after IVT [13]. In the DEVT trial, a similar effect was observed [14]. However, there was a wide range of noninferiority in that trial, which did not reach clinical consensus. In addition, all the enrolled subjects were endovascularly treated within 4.5 h; therefore, it is not possible to state the superiority or inferiority of the two treatment options beyond 4.5 h. Thus, it is still debatable whether EVT is beneficial for the prognosis of AIS patients with LVO compared with IVT+EVT in different periods.

To clarify whether IVT with an injection of rt-PA before bolus retrieval in different periods is beneficial to patient prognosis, a comparison of efficacy between the subgroups of EVT alone and IVT+EVT in different periods was performed. The results of this study may provide a basis for the preoperative evaluation and selection of treatment options in different periods.

## Methods

### Trial design and oversight

This was a multicenter retrospective study that mainly compared the effectiveness (clinical results, recanalization rate) and safety (intracranial hemorrhage, mortality) between EVT and IVT+EVT in the different periods of treatment time. The protocol was approved by the institutional review board.

### Patient selection

**Fig. 1:**
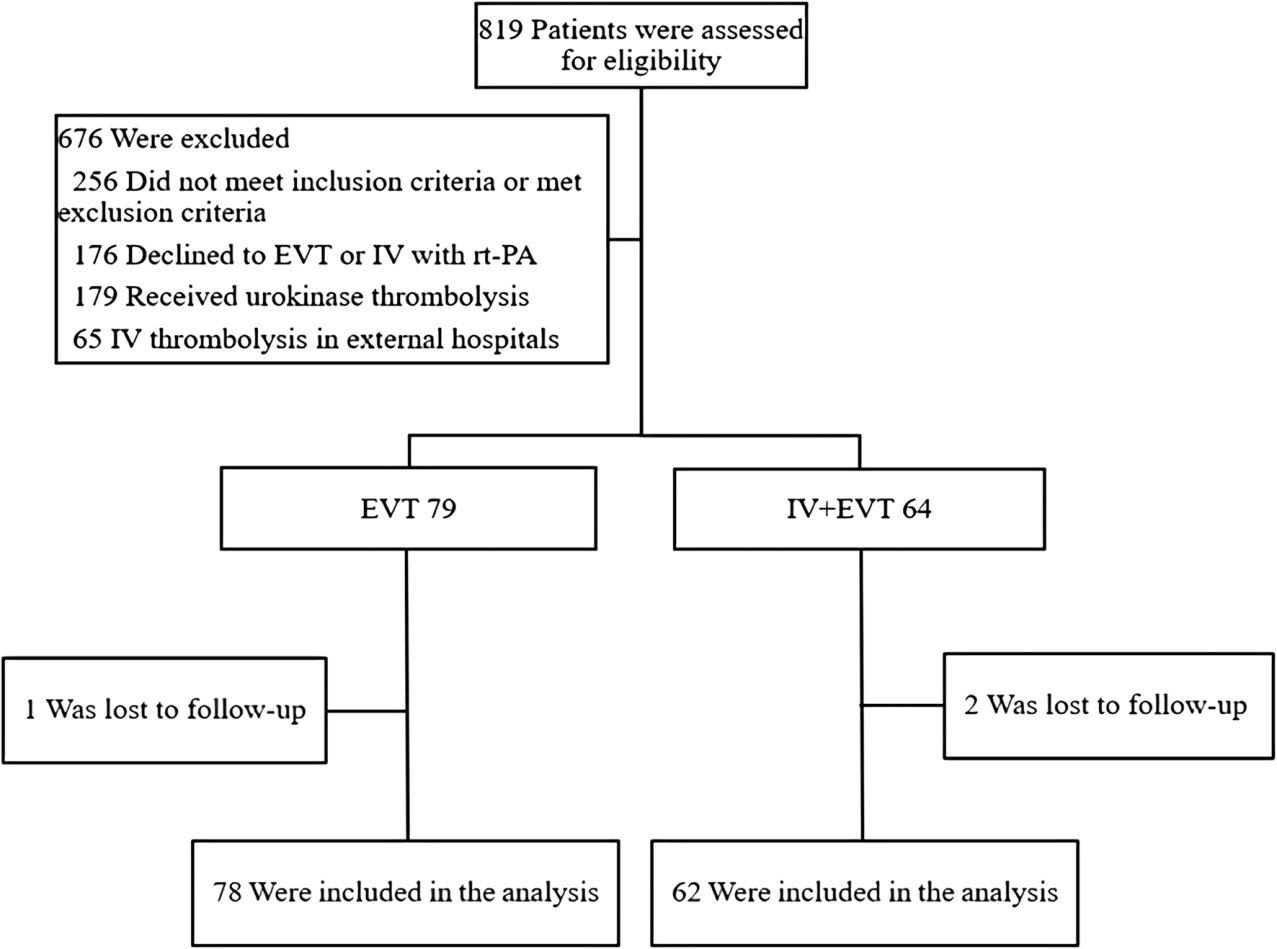
Flow chart of the analysis of the AIS patients in this study. EVT: endovascular therapy, IVT: intravenous thrombolysis

**Fig. 2:**
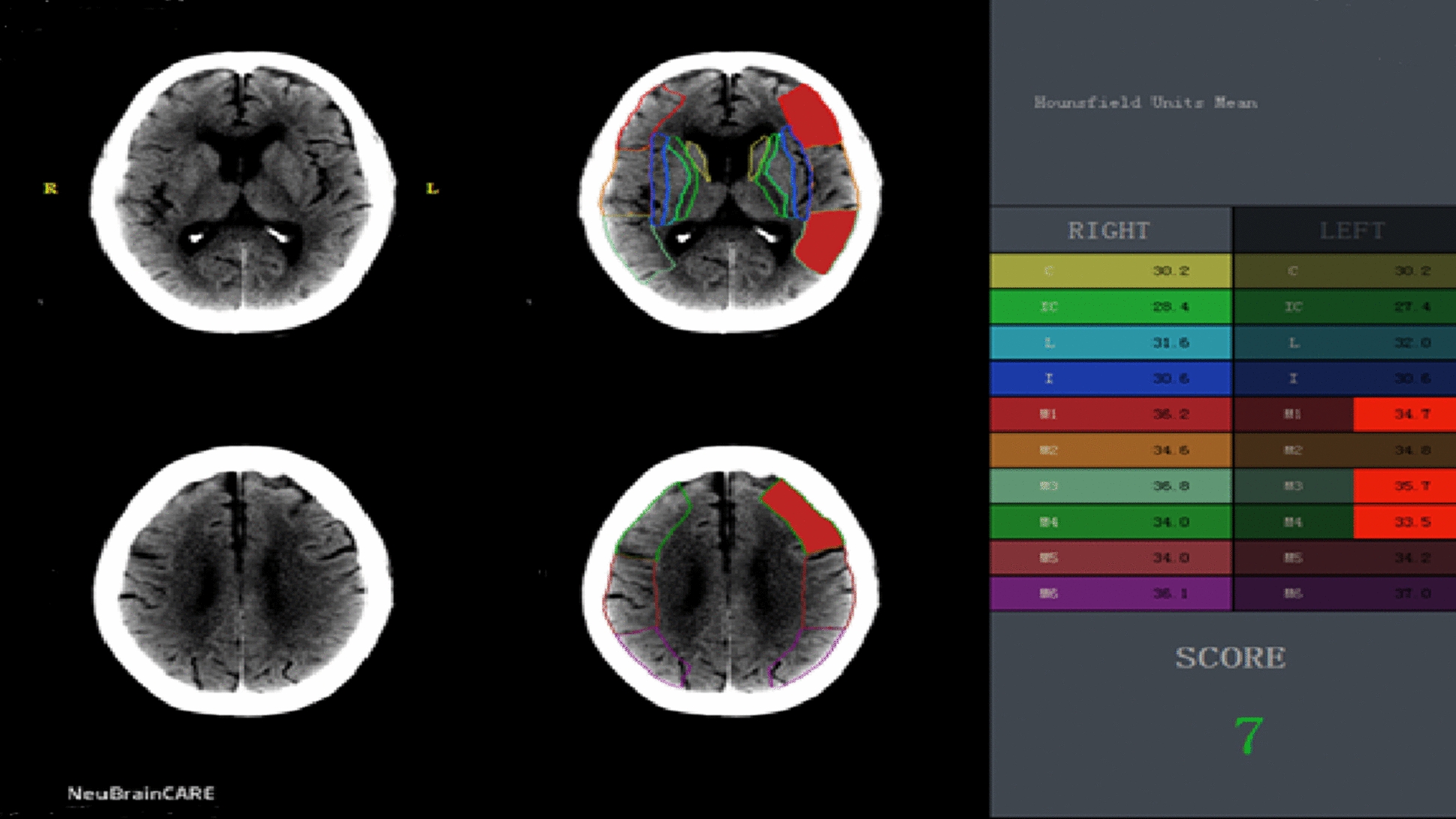
Automatic ASPECTS of CT plain scan images by NeuBrainCARE. The automatic ASPECTS was obtained after the correction and evaluation of CT plain scan images by analysis using NeuBrainCARE, which marks the red area as the affected area, with the automatic ASPECTS value (score 7)

We collected 140 AIS patients with LVO in the anterior circulation from three local advanced stroke centers between March 2019 and March 2022 (Fig. 1). The inclusion criteria of this study were as follows: (1) confirmed diagnosis of AIS; (2) age ranging between 18 and 80; (3) baseline National Institutes of Health Stroke Scale (NIHSS) score ≥ 4 or with isolated aphasia or hemianopia; (4) Alberta Stroke Program Early CT Score (ASPECTS) ≥ 6; (5) pre-onset Modified Rankin Scale (MRS) score ≤ 1; (6) vascular occlusion associated with neurological deficit; and (7) duration of symptoms less than 10 h [15]. The exclusion criteria were as follows: (1) age > 80 years; (2) incomplete data; (3) lost cases; (4) posterior circulation occlusion; and (5) received outside hospital thrombolysis. All patients in this study had intracranial occlusion of the internal carotid artery (ICA), middle cerebral artery M1 or M2, or both, all of which were confirmed by digital subtraction angiography. The IVT procedure in our study was performed according to international and institutional guidelines [4, 16]. In the IVT+EVT group, patients received IVT at a dose of 0.9 mg/kg body weight, before which rt-PA was given within 4.5 h after the onset of symptoms, according to the patient’s condition and other factors. However, the final treatment decision was made by an interdisciplinary team of neurologists and neuroradiologists based on the basic situation of the case. Therefore, in some cases, especially in the case of LVO suspected to have a large thrombus burden, EVT directly could be the first choice of the team, rather than IVT+EVT. The EVT procedure included the following aspects: stent thrombectomy, thrombus extraction, stent forming, balloon forming, and mechanical removal of blood clots with or without local use of the thrombolytic agent. The corresponding scores of the cases were calculated by analyses of the computed tomography (CT) image data. In this study, we used Neusoft Brain Clinical Assistant Ration Evaluate (NeuBrainCARE, China) to avoid the large infarct area of the included cases. NeuBrainCARE is a brain disease analysis software that focuses on quickly providing evidence of thrombolysis and EVT via the detection of blockages in major brain blood vessels [17], which can automatically partition the brain and calculate the ASPECTS on the CT sequence, thereby finding the cases with ASPECTS ≥ 6 to ensure the reliability of our study results (Fig. 2). When patients were discharged, they were asked to conduct a follow-up (90 days) in the outpatient department of the hospital to re-check the MRS score. For patients who did not visit the outpatient department in time after 90 days, we evaluated their MRS score through telephone follow-up. Patients transferred after thrombolysis in external hospitals were not included in our study.

### Study sites and interventionists

This study was performed at three advanced stroke centers in Shanxi Province. In those stroke centers, which are equipped with trained neurointerventional doctors, the number of hospitalized patients with AIS is more than 600 every year, and the number of cases with EVT and IVT+EVT is more than 200 each year.

### Evaluation of patient outcomes

The primary outcome for evaluation was the MRS score at 90 days after surgery based on a 7-level global disability measurement scale with scores ranging from 0 (asymptomatic) to 6 (death), including categories of good results (0–2) and bad results (3–6), according to the analysis of data collected from telephone follow-up or outpatient reexamination. Secondary outcomes included functional independence 90 days after surgery (MRS score ≤ 2), such as changes in the NIHSS score 24 h and 7 days after surgery, as well as vascular recanalization, which is defined by the score of the modified thrombolysis in cerebral infarction (mTICI) ranging from 0 to 3; mTICI ≥ 2b is considered to be successful recanalization. The safety outcome was also considered, which included the 90-day postoperative mortality and intracranial hemorrhage rate evaluated using the European Cooperative Acute Stroke Study-II standard [18, 19], in which parenchymal hematoma PH-1 or PH-2 was defined as significant bleeding.

### Statistical analyses

In this study, SPSS Statistics 22.0 software was used for data processing and data analysis. The chi-square test or Fisher’s exact probability method was used for comparisons among groups of categorical variables. The *t* test was used to compare the difference between groups for normally distributed measures, whereas the Mann–Whitney *U* test was used to compare between groups for non-normally distributed measures. At 90 days after treatment, the MRS scores in different subgroups were compared by ordered logistic regression analysis. The parameters in the multivariate model, including age, baseline NIHSS scores, ASPECTS, onset-to-revascularization time, and onset-to-puncture time, were also included for data analysis. We adjusted the results of the data analysis in this study according to theoretical knowledge, known relevant results, and empirical knowledge (baseline imbalance). Secondary outcomes were evaluated using logistic regression. *P* < 0.05 was considered statistically significant.

## Results

A total of 140 patients were enrolled in our study, including 78 who received EVT alone and 62 who received IVT+EVT. As shown in Table 1, the baseline characteristics of the patients in those two groups were similar, such as age, hypertension, diabetes, atrial fibrillation, current smoking status, ischemic stroke or transient ischemic attack history, and the ASPECTS. The mean age of the patients in the EVT alone group was 64 years, of whom 37 patients (52.4%) were male, and their median ASPECTS was 8 points (interquartile range [IQR] 6–10). The mean age of patients in the IVT+EVT group was 65 years, and similarly, 45 patients (74.5%) were male, and their median ASPECTS was also 8 points (IQR 6–10). The median time from onset to femoral artery puncture was 396 min (IQR 24–600) and from stroke onset to revascularization was 469 min (IQR 67–1140) in the EVT group, whereas in the IVT+EVT group, it was 271 min (IQR 43–600) and 380 min (IQR 140–731), respectively.

**Table 1:** Characteristics of the patients and workflow measures at baseline.*

Variable	EVT (*N* = 78)	IV + EVT (*N* = 62)	*p* Value
Mean age ± SD—yr	63.01 ± 11.42	65.29 ± 10.71	0.194
Male sex—no. (%)	37 (47.4)	45 (72.6)	0.024
Medical history—no. (%)			
Atrial fibrillation	27 (34.6)	17 (27.4)	0.113
Diabetes mellitus	10 (12.8)	12 (19.4)	0.128
Hypertension	37 (47.4)	30 (48.4)	0.819
Ischemic stroke or transient ischemic attack	19 (24.4)	8 (12.9)	0.233
Prior or current smoker	26 (33.3)	15 (24.2)	0.334
No symptoms on modified Rankin scale before stroke—no. (%)	21 (84.0)	30 (48.4)	0.064
NIHSS score†			
Median (IQR)	15 (4–39)	14 (4–32)	0.132
6–16—no. (%)	43 (55.1)	41 (66.1)	
17 or more—no. (%)	35 (44.9)	21 (33.9)	
Median systolic blood pressure at hospital arrival (IQR)—mmHg	136 (79–210)	141 (106–196)	0.194
Median glucose level at hospital arrival (IQR)—mmol/liter^‡^	6.11 (3.7–50.3)	5.83 (3.8–15.7)	0.695
Median ASPECTS value (IQR)§	8.0 (6.0–10.0)	8.0 (6.0–10.0)	0.069
Occlusion location, No. (%)			
ICA	25 (32.0)	20 (32.3)	0.876
M1 MCA	41 (52.6)	35 (56.5)	
M2 MCA	12 (15.4)	7 (11.2)	
Workflow times			
Median time from stroke onset to intravenous alteplase (IQR)—min	NA	176 (56–270)	
Median time from stroke onset to groin puncture (IQR)—min	396 (24–600)	271 (43–600)	0.002
Under 4.5 h—no. (%)	24 (30.7)	31 (50.0)	
4.5–6 h—no. (%)	10 (12.8)	15 (24.2)	
6–8 h—no. (%)	25 (32.1)	11 (17.7)	
8–10 h—no. (%)	19 (24.4)	5 (8.1)	
Median time from stroke onset to revascularization (IQR)—min	469 (67–1140)	380 (140–731)	0.009

^*^ICA denotes internal carotid artery, M1 MCA denotes the first segment of the middle cerebral artery, M2 MCA denotes the first segment of the middle cerebral artery, IQR interquartile range, and NA not applicable

^**†**^Scores on the National Institutes of Health Stroke Scale (NIHSS) range from 0 to 42, with higher scores indicating more severe neurologic deficit

^**§**^The Alberta Stroke Program Early CT Score (ASPECTS) ranges from 0 to 10, with higher scores indicating a smaller infarct core

^**‡**^To convert the values for glucose to millimoles per liter, divide by 0.05551

### Primary outcome

**Fig. 3:**
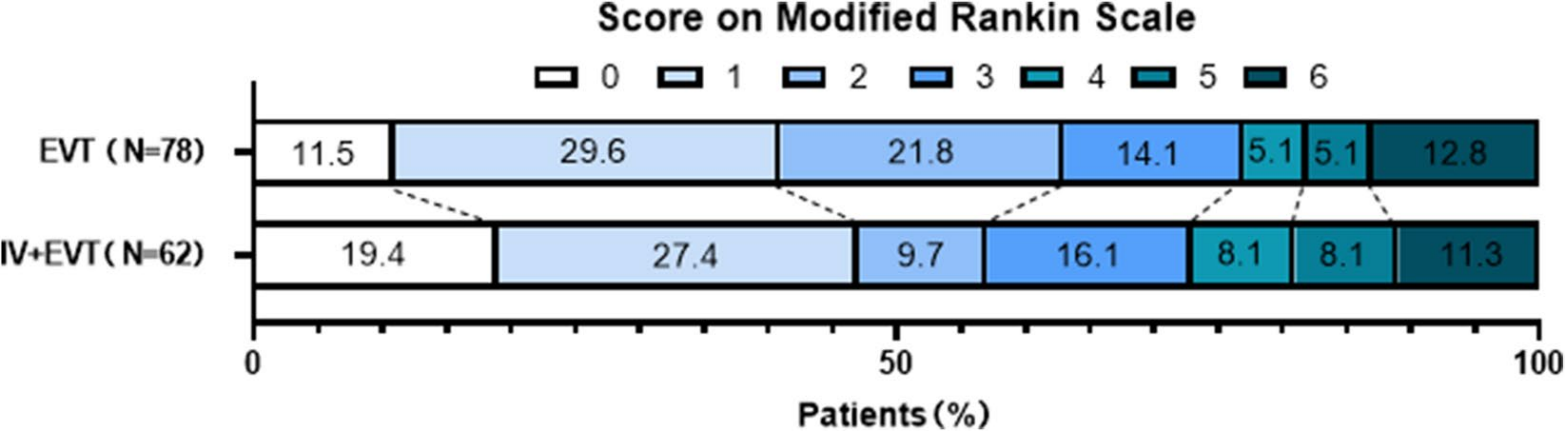
Scores on the MRS at 90 days in the intention-to-treat population. As shown in the picture, the percentages of patients in the endovascular therapy group and alteplase-only group with scores from 0 to 6, according to the MRS as follows: 0, no symptoms; 1. no clinically significant disability; 2. slight disability (able to handle own affairs without assistance but unable to carry out all previous activities); 3. moderate disability requiring some help (e.g., with shopping, cleaning, and finances but able to walk unassisted); 4. moderately severe disability (unable to attend to bodily needs without assistance and unable to walk unassisted); 5. severe disability (requiring constant nursing care and attention); and 6. death. Patients with a score of 0, 1, or 2 are considered to be independent in daily function

The evaluation of the primary outcome revealed that 49 cases (62.8%) in the EVT group had an MRS score of 0–2 at 90 days after the operation, whereas 35 cases (56.5%) in the IVT+EVT group had a similar MRS score (Fig. 3). Ninety days after the operation, the adjusted common odds ratio (OR) of the MRS score was 0.81 (95% confidence interval [CI] –0.86 to 0.43; *P* = 0.52) (Table 2). Therefore, there was no significant difference in MRS score between the EVT and IVT + EVT groups.

**Table 2:** Functional and safety outcomes for the entire cohort and subgroups

	EV-Only (*n* = 78)	IV + EV (*n* = 62)	OR(95% CI)		*p* Value
			Unadjusted	Adjusted	
Primary outcome					
mRS at 90 d Median score (IQR)	2 (0–6)	2 (0–6)	1.09 (− 0.50–0.68)	0.81 (− 0.86–0.43)	0.52
Secondary outcomes					
mRS 0–2 at 90 d—no./total no. (%)	29 (37.2)	27 (43.5)	1.30 (0.66–2.57)	1.72 (0.78–3.78)	0.18
Change in NIHSS score at 24 h Median (IQR)‡	2(− 30–24)	0 (− 24–16)			0.12
Change in NIHSS score at 7d Median (IQR)‡	7(− 30–26)	4 (− 24–23)			0.15
mTICI score of 2b or 3—no./total no. (%)	65 (83.3)	49 (79.0)	0.69 (0.29–1.59)	0.59 (0.23–1.55)	0.29
Safety outcomes					
Mortality at 90 d—no/total no. (%)	14 (17.9)	12 (19.4)	1.2 (0.50–2.86)	1.56 (0.58–4.21)	0.38
Hemorrhage—no./total no. (%)	2 (2.6)	7 (11.3)	4.8 (0.97–24.18)	8.21 (1.28–52.82)	0.03

### Secondary outcomes

The adjusted common OR for functional independence (MRS score ≤ 2) at 90 days after the operation was 1.72 (95% CI 0.78 to 3.78; *P* = 0.18). The NIHSS score was not significantly different between admission and postoperation at 24 h (*P* = 0.12) or 7 days (*P* = 0.15). The adjusted common OR of the postoperative recanalization rate was 0.59 (95% CI 0.23 to 1.55; *P* = 0.29). Successful revascularization was achieved in 83.3% of patients in the EVT alone group and in 79.0% of patients in the IVT+EVT group (Table 3). The abovementioned results revealed that there was no statistically significant difference in secondary outcomes between the two treatment groups. In the 6–8 h subgroup, the NIHSS score was significantly different between admission and postoperation at 24 h (*P* = 0.01) or 7 days (*P* = 0.02). The analyses of other subgroups are shown in Table 4.

**Table 3:** Scores on the modified thrombolysis in cerebral infarction scale for 78 patients in the thrombectomy group and 62 patients in the bridging treatment group

**Score**	At baseline		After treatment	
	No. of patients (%)			
	EVT	IVT+EVT	EVT	IVT+EVT
0: No reperfusion	47 (60.3)	29 (46.7)	2 (2.6)	0
1: Antegrade flow past the initial occlusion but limited distal branch filling with little or slow distal reperfusion	22 (28.2)	19 (30.6)	5 (6.4)	3 (4.8)
2a: Antegrade reperfusion of less than 50% of the previously ischemic territory	6 (7.7)	10 (16.1)	6 (7.7)	11 (17.7)
2b: Antegrade reperfusion of more than 50% of the previously ischemic territory	3 (3.8)	4 (6.5)	18 (23.0)	18 (29.1)
3: Complete antegrade reperfusion of the previously ischemic territory, in absence of visualized occlusion in all distal branches	0	0	47 (60.3)	30 (48.4)

**Table 4:** Functional and safety outcomes of different subgroups

	under4.5 h			4.5–6 h			6–8 h			8–10 h		
	EVT (*n* = 24)	IV+EVT (*n* = 31)	*P*	EVT (*n* = 10)	IV+EVT (*n* = 15)	*P*	EVT (*n* = 25)	IV+EVT (*n* = 11)	*P*	EVT (*n* = 19)	IV + EVT (*n* = 5)	*P*
Primary outcome												
mRS at 90 d Median score (IQR)	2 (0–6)	2 (0–6)		2 (0–6)	2 (0–5)		2 (0–6)	3 (0–6)		2 (0–6)	2 (1–3)	
Secondary outcomes												
mRS 0–2 at 90 d—no./total no. (%)†	9 (37.5)	6 (19.4)	0.74	3 (30.0)	13 (86.7)	0.47	8 (32.0)	6 (54.5)	0.18	9 (47.4)	2 (40.0)	0.59
Change in NIHSS score at 24 h Median (IQR)‡	3 (− 6–24)	0 (− 18–16)	0.06	0 (− 30–21)	0 (− 30–21)	0.58	5 (− 3–19)	0 (− 18–6)	0.01	0 (− 14–14)	5(-6–5)	0.14
Change in NIHSS score at 7d Median (IQR)‡	8 (− 4–22)	4 (− 24–23)	0.45	2 (− 30–21)	4 (− 30–21)	0.80	9 (− 3–26)	2 (0–10)	0.02	3 (− 28–14)	6 (− 8–9)	NA
mTICI score of 2b or 3—no./total no. (%)	23 (95.8)	23 (74.2)	0.03	8 (80.0)	12 (80.0)	0.70	22 (88.0)	9 (81.8)	0.25	12 (63.2)	5 (100)	0.15
Safety outcomes												
Mortality at 90 d—no/total no. (%)	6 (25.0)	6 (19.4)	0.62	2 (20.0)	2 (13.3)	0.53	1 (4.0)	4 (36.4)	0.02	5 (26.3)	0	NA
Hemorrhage—no/total no. (%)	1 (4.2)	2 (6.5)	0.60	0	2 (13.3)	0.35	1 (4.0)	2 (18.2)	0.22	0	1 (20.0)	NA

Favorable reperfusion was defined as reperfusion of at least 50% and a modified Thrombolysis in Cerebral Infarction score of 2b (50–99% reperfusion) or 3 (complete reperfusion), as assessed with the use of DSA

^*^The MRS of functional disability ranges from 0 (no symptoms) to 6 (death)

^†^Functional independence was defined as a score of 0, 1, or 2 on the MRS

^‡^Change in NIHSS score was defined as a difference value of the NIHSS score between admission and postoperation at 24 h. NIHSS scores at 24 h or in 7 days

### Safety outcomes

During the 90-day follow-up, the adjusted common OR of postoperative mortality in the two treatment groups was 1.56 (95% CI 0.58 to 4.21; *P* = 0.38). The adjusted common OR of the postoperative intracranial hemorrhage rate in the two treatment groups was 8.21 (95% CI 1.28 to 52.82; *P* = 0.03), indicating that there was no significant difference in mortality between the two treatment groups. Moreover, IVT+EVT might increase the risk of intracranial hemorrhage. In the 6–8 h subgroup, the mortality (*P* = 0.02) was significantly different between groups. The analyses of other subgroups are shown in Table 4.

## Discussion

In our study, the comparison of clinical outcomes showed that there was no difference in recanalization rate, mortality, or long-term functional outcomes in stroke patients who received rt-PA thrombolysis before EVT compared to those in patients who received EVT alone, except for the increased risk of intracranial hemorrhage. However, in the 6–8 h subgroup, the effect of EVT alone was not inferior to that of IVT+EVT, whereas the DEVT study showed no significant difference between the two groups regarding mortality and incidence of spontaneous cerebral hemorrhage [14], which may be due to the exclusion of patients with M2 segment occlusion in DEVT. Tsivgoulisg et al. [20] reported that the efficacy of EVT in acute LVO does not depend on IVT pretreatment, according to a systematic review and meta-analysis of all available randomized controlled trials. A meta-analysis by Chen et al. [21]*.* suggested that in patients with anterior circulation AIS within 4.5 h after onset, EVT was not inferior to IVT therapy in combination with intravascular EVT. Weber [22] and others believe that in patients with AIS treated with EVT, the use of IVT is not an independent predictor of a good prognosis, because there is no difference in the incidence of complications whether IVT is used. However, the study included cases with pre-stroke MRS ≥ 3 and with severe comorbidities, which may have impacted the reliability of the results. A retrospective study [23] showed that EVT combined with IVT within 4.5–9 h after onset might be safe in patients with anterior circulation artery occlusion due to the lack of a significantly increased risk of intracranial hemorrhage. Our results are not consistent with the abovementioned study. In our study, the subjects in the IV+EVT group were intravenously thrombolyzed within 4.5 h, suggesting an increased risk of intracranial hemorrhage when intravenous thrombolysis was given before endovascular treatment. Based on clinical outcomes, such as the improvement of neurological function 24 h and 7 days after the operation as well as the mortality (90 days) after the operation, the efficacy of EVT in the 6–8 h subgroup was better than that in the same subgroup of IVT+EVT. However, there was no significant difference between those two treatments in the other subgroups.

However, due to the relatively small sample size in this study, especially in the 8–10 h subgroup, the reliability of the data might be reduced. Therefore, it is still unclear whether there will be differences in the 8–10 h subgroup with an increase in sample size, even including the differences between the two treatments after 6 h, which needs a larger sample size to confirm. Using current evidence, it is recommended that EVT be directly performed within 10 h after the onset of the disease, but IVT is not necessary. However, within this time window, the effectiveness of the treatment of IVT+EVT or EVT needs to be verified in larger randomized controlled trials.

Park [24] and others reported a prospective multicenter stroke registration database in Korea that included 639 AIS patients, of whom 458 received IVT before EVT. That report showed that giving IVT before EVT within 8 h after the onset of AIS could improve both the survival rate and recanalization rate without increasing the risk of symptomatic hemorrhage, leading to a decrease in disability symptoms 3 months after treatment. That report revealed that IVT is beneficial before endovascular treatment, which is inconsistent with our results. It is notable that the subjects enrolled in the abovementioned study also included those with posterior circulation AIS, which may be the reason why their findings are not consistent with our results.

Moreover, in some AIS patients with LVO, IVT before EVT may have several disadvantages, such as poor migration of embolus and an increase in the following aspects: ischemic area, the risk of intracranial hemorrhage, medical consumption, and the burden of patients. Administration of IVT before EVT may also delay the duration time from onset to femoral artery puncture, especially in the case of AIS patients with LVO who must be transferred to the comprehensive stroke center for further treatment after receiving intravenous injection in a local stroke center, where there are no EVT conditions [25]. Among the cases we collected, 65 patients were excluded due to a delayed time from onset to femoral artery puncture, because those patients were transferred to our hospital after receiving IVT in a lower level hospital without EVT conditions.

Despite its shortcomings, IVT can promote either intravascular reperfusion or the dissolution of microemboli lodged in the downstream precapillary vasculature and can also improve distal perfusion [26, 27]. The benefit of IVT therapy should not be ignored. It is important for clinicians to perform IVT in AIS patients who have no contraindications to thrombolysis within the time window.

Accordingly, we conducted EVT treatment in patients with acute anterior circulation ischemic stroke within 10 h from symptom onset to femoral artery puncture. The EVT was proven safe compared to the IVT+EVT, especially for patients with acute anterior circulation ischemic stroke within 6–8 h from symptom onset to femoral artery puncture. Thus, EVT can not only promote the recovery of neurological function but can also decrease the risk of intracranial hemorrhage.

This study had some limitations. First, this retrospective study was prone to selection bias. Second, we excluded some cases with contraindications to thrombolysis from the EVT group, resulting in a small sample size, which may have affected the reliability of our results. In previously reported studies, a paired analysis study based on two large registrations (no thrombolytic contraindications in the EVT group) showed that except for the high mortality of patients with ICA occlusion, there was no difference in the outcome of IVT patients with LVO anterior circulation stroke when EVT alone and IVT+EVT for those patients were compared [28]. That study could support the reliability of our results to some extent. Finally, compared to many Western countries, China’s prehospital triage system is more complex, where patients usually go to the hospital directly by private transportation; in such a situation, the stroke treatment team is usually mobilized only when the patient is admitted to the hospital, rather than before the patient arrives [13]. Moreover, most of the patients originally came from remote areas, and their families and patients themselves had limited knowledge about stroke. Therefore, after the onset of stroke, they cannot go to the hospital immediately. Even if they arrive at the hospital, timely treatment can also be delayed due to some preparation steps, such as talking to family members of the patient and providing signed informed consent before rt-PA use and thrombus removal. Similarly, the time interval from onset to femoral artery puncture also varied in our study. In some cases, the time interval was significantly greater than 10 h, leading to a small sample size in the subgroups, which may have had a certain impact on the reliability of our results.

In conclusion, our study provides a basis for the selection of treatment options in patients with acute anterior circulation ischemic stroke within 10 h after the onset of symptoms.

## Data Availability

The data sets used or analyzed during the current study are available from the corresponding author on reasonable request.
